# Equal Opportunities for Stroke Survivors’ Rehabilitation: A Study on the Validity of the Upper Extremity Fugl-Meyer Assessment Scale Translated and Adapted into Romanian

**DOI:** 10.3390/medicina56080409

**Published:** 2020-08-13

**Authors:** Nadinne Roman, Roxana Miclaus, Angela Repanovici, Cristina Nicolau

**Affiliations:** 1 Faculty of Medicine, Transilvania University of Brasov, 500019 Brasov, Romania; nadinneroman@unitbv.ro; 2 Faculty of Product Design and Environment, Transilvania University of Brasov, 500068 Brasov, Romania; arepanovici@unitbv.ro; 3 Faculty of Economic Sciences and Business Administration, Transilvania University of Brasov, 500068 Brasov, Romania; cristina.nicolau@unitbv.ro

**Keywords:** rehabilitation, assessment, physiotherapy, clinical evaluation

## Abstract

*Background and objectives:* The Upper Extremity Fugl-Meyer Assessment (UEFMA) is one of the most recommended and used methods of clinical evaluation not only for post-stroke motor function disability conditions but also for physiotherapy goal-setting. Up to the present, an official Romanian version has not been officially available. This study aims to carry out a translation, adaptation, and validation of UEFMA in Romanian, thus giving both patients and medical practitioners the equal opportunity of benefiting from its proficiency. *Material and methods:* The English version of the motor component of UEFMA was back and forth translated in the assent of best practice translation guidelines. The research was performed on a group of 64 post-stroke in-patients regarding psychometric properties for content validation and an exploratory and confirmatory factorial analysis was performed using the Bayesian model. To assess internal consistency and test–retest reliability, we used the Cronbach Alpha index and Intraclass Correlation Coefficient (ICC). We used Pearson correlation with the Functional Independence Measure (FIM) and Modified Rankin Scale (MRS) to determine concurrent validation. Standardized response mean (SRM) was applied to determine the responsiveness of the instrument used. *Results:* After performing the exploratory factor analysis, a single factor was extracted, with an Eigenvalue of 19.363, which explained 64.543% of the variation. The model was confirmed by Bayesian exploration, with Root Mean Square Residual (RMR) 0.051, Goodness-of-fit Index (GFI) 0.980, Normed-Fit Index (NFI) 0.978 and Relative Fit Index (RFI) 0.977. The Cronbach Alpha value was 0.981, the Intraclass Correlation Coefficient (ICC) index for average measures was 0.992, the Pearson correlation with FIM 0.789, and MRS −0.787, while the SRM was 1.117. *Conclusions:* The Romanian version of the UEFMA scale is a reliable, responsive and valid tool which can be used as a standardized assessment in post-stroke patients across Romania.

## 1. Introduction

Stroke is the third leading cause of disability worldwide. Over the past decades, low- and middle-income countries have doubled their stroke-caused incidence of morbidity and disability, whereas high-income countries registered a decrease of 42% [1]. Concerning physical rehabilitation of patients with stroke sequelae, they need adequate clinical and functional assessment. A rigorous and adequate evaluation is essential both for the rehabilitation physician and physiotherapists for monitoring patients’ evolution, prescribing an adequate drug treatment, and also for establishing physiotherapy and physical recovery outcomes/goals. [2,3]. Globally, a large number of assessment evaluation scales have been developed to determine: the level of disability or functional independence (Barthel scale, Functional Independence Measure (FIM) or Activities of Daily Living (ADL) scale, Modified Rankin Scale (MRS)), upper and lower extremities motor function, balance, cognitive function and speech, stroke severity, the somatosensory function (Nottingham scale), spasticity (Ashworth scale) or depression (Post Stroke Depression Rating Scale, PSDRS scale) [4,5,6,7,8,9].

Concerning developing countries, which may often encounter a lack of resources, the use of appropriate tools to assess stroke patients’ physical status is of significant importance in three aspects: (a) for modelling the selection criteria for rehabilitation; (b) implementing the proper strategies to efficiently concentrate human, logistical and financial resources at patients irrespective of the probabilities of rehabilitation, social and even professional integration; (c) selecting, using and tailoring physiotherapy protocols according to patients’ degrees of disability and by difficulty levels [10].

A large number of international guides and other research conducted in the field suggest that the Upper Extremity Fugl-Meyer Assessment (UEFMA) is a valid instrument given its excellent psychometric properties and suitable scale for assessing the functionality and the motor function of the post-stroke upper extremity (UE). Additionally, we underline that apart from the benefits that research regarding the use of UEFMA has shown, it was also validated using virtual reality technology, more precisely through the Kinect sensor [11,12,13].

Initially, the UEFMA was developed by Fugl-Meyer for the assessment of motor function, balance, sensitivity, and joint mobility. The entire version has 113 items, while the subscale for UE assessment examines 63 elements (55.75%). In regards to UE feature, 33 elements (29.20%) evaluate motor function, six items (5.31%) assess sensitivity and proprioception, while the last 24 points (38.09%) rate joint pain and mobility. Every item of the assessment scale is marked on an ordinal level, from 0 to 2; the 0 value corresponds with the impossibility to perform a movement, and 2 represents the ability to perform a complete and adequate movement [14]. However, the psychometric properties of the balance and somatosensory evaluation subscales, such as their validity and reliability, have proved to be questionable, not being as effective as the other constitutive parts [15,16].

Regarding the UE assessment as a particular constitutive part in the case of patients in subacute and chronic post-stroke stages, subsequent research has reported significant inferences with/matching to other similar assessment scales, such as the Action Research Arm Test and the Wolf Motor Function Test [17,18]. In regards to the construct validity of the motor function evaluation, research, including a Rasch analysis, has shown that the three items referring to the reflex activity did not significantly contribute to the evaluation of the UE, thus determining the extraction of the three main factors and consequently, UEFMA’s reduction to 30 items [19,20].

Like many other assessment scales that are translated from English and adapted into the national language [21,22,23,24,25], originally in English and Swedish, up to the present UEFMA has been translated and used in Italy, Japan, The Netherlands and the USA [26] and has been validated in its translated forms for Danish and Spanish (Colombian) [27,28].

Under such circumstances, our research aimed to translate, adapt and validate the UEFMA into Romanian, as well as to establish the test–retest reliability and concurrent validity, since the instrument is reliable and is used at an international level. Consequently, this may allow data analysis on post-stroke rehabilitation in different countries and regions, with subsequent worldwide implications on physical post-stroke rehabilitation [29].

## 2. Materials and Methods

### 2.1. Study Design

The translation into Romanian of the instrument used and its cultural adaptation was performed according to the standard backward and forward translation procedures to determine the concept and technical equivalents [30]. The UEFMA scale was translated from English into Romanian by two independent translators, each with an advanced level of English. One of the translators was familiar with the field and its medical terms, whereas the second translator did not know medical terminology. A third translator participated in the synthesis of the two translated versions, knowing both the medical language and the usual spoken language, and elaborated a single consensual version. Later, a translation was made from Romanian into English by an English teacher. The translated version from Romanian into English and the original English version were compared by another native English speaker to assess whether the text retained its original meaning.

The preliminary version of the assessment scale was initially tested and applied by five physiotherapists, followed by a focus group interview and discussions of understanding the elements and the consensus. The focus group also determined whether there were inconsistencies in the wording, which could negatively or positively influence the scoring, understanding, interpretation and cultural application. We took into account the specific features of the Romanian language so that the scale could be understandable for medical professionals and could be easily applied to patients.

In our paper, for each item of the UEFMA scale, we used the index and numbering from the original version. All the elements and subpoints from A to D, regarding the motor function of the UE assessment, are presented. After we applied the described procedure regarding back and forward translation, and also the focus group interview with the physiotherapists, we made changes in the assessment scale within AII, AIV, B3, and B4 items from the initial version.

In regards to the AII item, within the flexor’s synergy, we considered that an explanation was needed, so we added the words “with palm upward” after the phrase “hand from contralateral knee to the ipsilateral ear”. We have made these adjustments so that neither any future discrepancy between the evaluators using this instrument nor any incorrect evaluation of the supination to be performed will occur.

Regarding AIV3 item which assesses the pronation-supination movement, we agreed to use the value of 30–40 degrees for shoulder flexion where ”shoulder at 30°–90° flexion” occurs in the original text. Within the B3 and B4 items which describe the UE initial position ”with the elbow at 0° and slight shoulder flexion/abduction”, we replaced ”slight shoulder flexion/abduction” with “shoulder flexion/abduction of 20–30°”.

### 2.2. Study Participants

The study was conducted in the Clinical Hospital of Psychiatry and Neurology in Brașov, Romania, from July to December 2019, which allowed us to recruit our participants. The research bears the approval of the before mentioned hospital under No. 12534/7 July 2019. Participants gave their written informed consent according to the present legislation and medical research ethics. We underlined to our participants that they could cancel their participation in the study during their hospitalization, but also that they subsequently might withdraw their agreement according to which we used the clinical data provided. Whatsoever, no personal data were used.

We included in our sample patients with post-stroke subacute or chronic hemiparesis, at least six weeks post-stroke. As exclusion criteria, we applied these for patients with an unstable medical condition, severe cognitive dysfunctions, and receptive aphasia. In total, 64 post-stroke patients participated in the research.

Two trained physiotherapists assessed the group of patients using the Romanian version of UEFMA (see Supplementary Materials): firstly, we collected data at the beginning of patients’ hospitalization and finally, at their discharge after 14 days.

### 2.3. Data Analysis

We analyzed the data using the Statistical Package for the Social Sciences (IBM SPSS Statistics for Windows, Version 20.0. Armonk, NY: IBM Corp.) software version 20, and we performed the structural equation modelling (SEM) using Amos (Version 26.0), Chicago: IBM SPSS.We performed an exploratory factor analysis (EFA) initially, using the Principal Axis Factoring as the extraction method and Quartimax rotation with Kaiser Normalization. We used the Quartimax rotation because it maximizes the sum of squares of the coefficients between the resulting vectors for each of the primary variables [31]. To verify if the data pass the assumptions, we explored the correlations matrix, as all the variables should correlate with at least another variable, with *r* ≥ 0.3. We checked for the values of Kaiser–Meyer–Olkin (KMO) and the Bartlett sphericity test, where KMO > 0.5 and Bartlett *p* < 0.05 are considered as proper values for EFA [32].

We conducted a confirmatory factor analysis (CFA) using structural equation modelling (SEM) and unweighted least square method for estimates calculation, since the maximum likelihood estimators are not an appropriate method for ordinal variables, such as in the case of UEFMA. [33] The following indexes of adherence of the model were used: Root Mean Square Residual (RMR) and Goodness-of-fit index (GFI), Normed-Fit Index (NFI) and Relative Fit Index (RFI) for baseline comparison, and Parsimonious Normed Fit Index (PNFI) as parsimony measures. The indices values are considered that the model fits if RFI ≥ 0.9, GFI and NFI ≥ 0.95, RMR< 0.08, while PNFI ≥ 0.80 [34].

Additionally, we used Cronbach Alpha to determine internal consistency and Intraclass Correlation Coefficient (ICC) for test–retest reliability. To examine concurrent validity, we used the Pearson correlation with Functional Independence Measure (FIM) and the Modified Rankin Scale (MRS). We used the standardized response mean (SRM) to test responsiveness.

## 3. Results

Firstly, we present the sample characteristics. The mean age of the participants was 59.76 years (standard deviation, SD = 8.53) with a minimum of 36 years and a maximum of 73 years. The mean time since stroke was 34.10 weeks (SD 38.04) with a minimum of 6 weeks and a maximum of 126 weeks (2.4 years). In the total sample, 43.75% (*n* = 28) of participants had a right-sided stroke (left hemiparesis) and 56.25% (36) had a left-sided stroke (right hemiparesis). Out of the 64 subjects, 46.87% (*n* = 30) were women and 53.13% (*n* = 34) were men.

Secondly, the KMO value of 0.913 indicates that the data used were fit for the EFA, with χ^2^ of Bartlett’s sphericity test of 2648.235 and *p* < 0.001. Initially, after applying the EFA, four factors with Eigenvalue surpassing one were extracted in the unrotated solution. Upon inspection of the Scree Plot and data related to the variation of every factor, we observed that the first factor had an Eigenvalue of 19.337, explaining 64.456% of the variation. In the rotated factor matrix results, all variables loaded on only one factor. The data obtained together with the inspection of the Scree Plot led us to retain only one factor and to consider the unidimensionality of the scale. The results obtained in the communalities values and the variables loading in the factor resulting from the EFA are presented in Table 1. 

The value of Cronbach Alpha indicates a high internal consistency. The ICC value suggests an excellent correlation between the initial and the final evaluation, while the Pearson correlation index shows a significant correlation with Functional Independence Measure (FIM) and Modified Rankin Scale (MRS). Furthermore, the responsiveness of the instrument used is high. The results are presented in Table 2.

**Figure 1:**
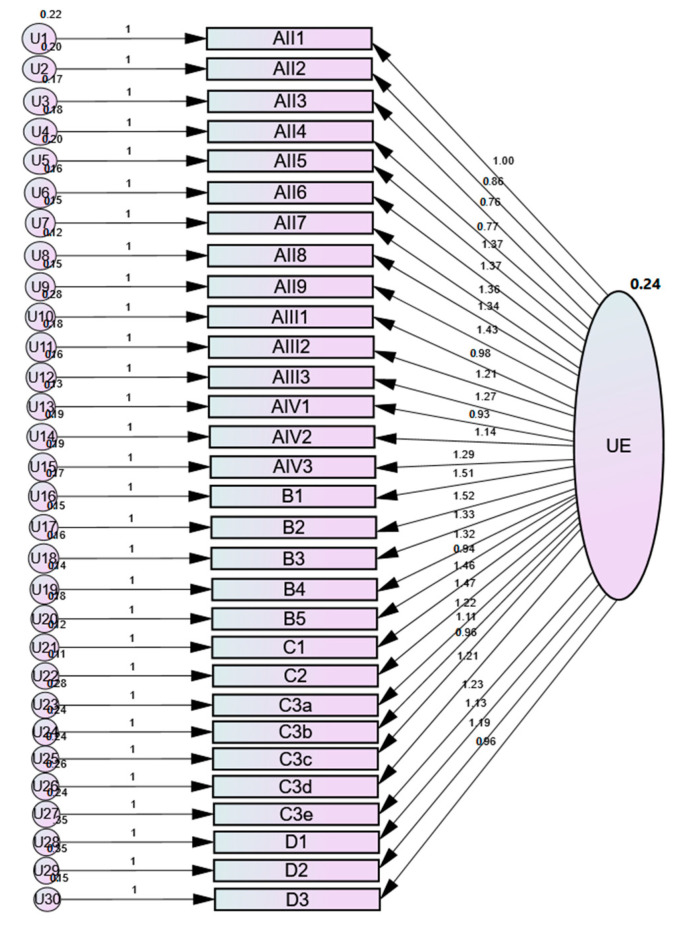
CFA results of UEFMA with unweighted least square method.

In Table 3 the coefficients of the model fit for CFA are presented, which was conducted secondary to EFA. Figure 1 represents the description of the CFA, which confirms the unidimensionality of the assessment scale.

Table 4 display the values obtained from the Bayesian modelling, with the regression weight values. The values of the intercepts and the variation are attached to additional files. The convergence value was set to 1.002 and the results were obtained in (500 + 61,501) × 16 iterations.

## 4. Discussion

Previous research on UEFMA and its constitutive part assessing motor function and the validity of the construct concluded that the items related to reflexes do not integrate into the construct of this assessment scale. Thus, the removal of these items made the evaluation tool herein analyzed as one-dimensional. Furthermore, in the first validation of the unidimensionality of the UEFMA, when performing PCA, initially four factors with Eigenvalue >1 were extracted, and only one factor explained 68% of the variation [19]. In this respect, the results of our study correlate with previous research and strengthen the unidimensionality of the instrument used in the assessment of UE motor function and functionality.

Regarding the value of the coefficients obtained with EFA, the weakest factor loadings were the external rotation of the shoulder (AII4), thumb’s opposition (pincer grasp, opposition) (C3c), shoulder abduction to 90 degrees (AII3), tremor (D1) and hand to the lumbar spine (AIII1). In CFA with Bayesian modelling, the lowest median regression weights were the factors related to 90-degree shoulder abduction (AII3), shoulder elevation (AII2) and shoulder external rotation (AII4) which confirm that the Bayesian ordinal CFA modelling is more robust than the EFA [33]. The results can also be explained by the fact that the patients in the study group were in their post-stroke subacute or chronic stages which usually implies that there are power and functionality at the proximal level of the upper limb, i.e., at the shoulder level.

Following the use of UEFMA at the beginning and end of physiotherapy, we obtained similar results with previous research using ICC, and this fact suggests that the translation and cultural adaptation of the UEFM scale in Romanian was performed properly. [29,35]. The internal consistency measured by the Cronbach Alpha index is also increased, by values close to previous results related to the psychometric properties of UEFMA, which strengthens the results obtained in our study on the instruments used [36,37]. The test–retest reliability, the concurrent reliability and the responsivity outcomes of the translated and adapted instrument in our study validate its accuracy [17].

Secondary to the translation and adaptation of the UEFMA scale into Romanian, our study can be used as a model for the translation, adaptation and validation of a clinical evaluation tool. We advocate for the rigor with which the research was performed, not only by using the information translation and adaptation guides present in the literature but also by following a rigorous and viable statistical analysis as a method of confirming the results. [27,30,38].

As in the case of the translation, adaptation and validation of UEFMA in Spanish, Danish and Japanese, it is proven that the UEFMA translated tool and used in our research is valid and can be used in Romanian in future studies [27,28,35]. Currently, for the evaluation of the upper extremity, a variety of evaluation tools are used, most of which are found in the recommendations of the international guides: Action Research Arm Test, Abilhand, Box and Block Test and Nine Hole Peg for dexterity; Chedoke Arm Hand Inventory, Frenchay Arm Test, UEFMA, Upper Limb Impairment Scale, Wolf Motor Function Test for upper extremity functional and motor assessment; active range of motion assessment (AROM) and muscle strength assessment through manual muscle testing (MMT) [5,11,12]. In addition to these upper extremity assessment scales, a correct patient clinical evaluation should also include the assessment of the lower limb, ADLs, gait, balance, the risk of falling, sensitivity and proprioception. However, researchers and clinicians alike should reduce time-consuming activities such the use of several evaluation tools that measure more or less the same items, with no well-standardized tool available [26].

The significance of translating and adapting clinical assessment scales at an international level is an essential factor in the development of research and the creation of a concise (synthesized) framework for the perception of diseases, disabilities and quality of life according to demographic, geographical and sociological factors [39]. In regards to rehabilitation and recovery, it is also a useful tool in the hands of clinicians and physiotherapists. In general, literature reviews and international guides seek to establish the most reliable assessment scales to facilitate medical practice and to standardize the assessment tools used in research. Overall, the UEFMA is part of both the recommendations of the international guides and the reviews alike, especially when considering that over the last few years and from a multitude of evaluation scales, the FMA has gained more and more ground as a tool used in research [11,12,26].

Furthermore, the lack of a standardized and internationally usable scale for post-stroke assessment, without different shortcomings of psychometric properties, led UEFMA to be successfully adapted and integrated, including in the virtual reality technology used in the physical rehabilitation of the patient, as a method of objective assessment [40]. Therefore, we consider that the translation, adaptation and validation of UEFMA is a step forward in its objective use, in clinical practice and research, but also as an integral part of the technology used in post-stroke neuro-rehabilitation, making uniform the assessment at a regional or global level. The need to integrate validated clinical scales into robotic technology and virtual reality technology developed for physical recovery is increasingly imperative under the circumstances of the new technologies mainly used in neuro-rehabilitation, which usually have assessment tools unrelated to the clinical assessment scales [41].

## 5. Conclusions

The Romanian version of UEFMA is a reliable, responsive and valid tool for measuring the motor function and functionality of the upper limb in the case of stroke survivors. As previously mentioned, its successful validation and cultural adaptation brings to rehabilitation practitioners an encouraging instrument to use within their future clinical or research activity. It also helps in predicting more clearly a possible rehabilitation period and makes possible the monitoring of post-stroke patients. Future work should further explore feasible assessment scales that can be translated and validated in different languages, for the uniformization of locomotory disabilities evaluation at a global level.

## Figures and Tables

**Table 1:** EFA (exploratory factor analysis) communalities and rotated factor matrix loading values for UEFMA (Upper Extremity Fugl-Meyer Assessment) items.

Communalities			Rotated Factor Matrix	
	Initial	Extraction	Factor	1
AII1	0.947	0.849	C2	0.907
AII2	0.917	0.756	B2	0.901
AII3	0.860	0.732	C1	0.890
AII4	0.823	0.675	B1	0.887
AII5	0.903	0.826	AII.8	0.879
AII6	0.922	0.785	B2	0.878
AII7	0.966	0.955	AII.9	0.869
90 AII8	0.937	0.842	B3	0.861
AII9	0.970	0.941	AII.7	0.852
AIII 1	0.869	0.649	AII.6	0.847
AIII 2	0.918	0.754	AII.5	0.832
AIII 3	0.895	0.744	AIV.3	0.828
AIV1	0.898	0.763	AIII. 3	0.824
AIV2	0.929	0.777	AIII.2	0.817
AIV3	0.858	0.740	AIV.2	0.814
B1	0.926	0.820	AIV.1	0.808
B2	0.945	0.888	D3	0.769
B3	0.905	0.801	C3e	0.768
B4	0.927	0.832	C3d	0.751
B5	0.750	0.576	AII.1	0.749
C1	0.957	0.895	C3b	0.746
C2	0.956	0.891	C3a	0.745
C3a	0.877	0.813	B5	0.744
C3b	0.937	0.808	D2	0.719
C3c	0.850	0.708	AII.2	0.710
C3d	0.936	0.862	AIII. 1	0.698
C3e	0.944	0.805	D1	0.696
D1	0.866	0.652	AII.3	0.695
D2	0.902	0.680	C3c	0.683
D3	0.815	0.692	AII.4	0.680

Note: Each item corresponds to the numbering on the initial UEFMA scale in English.

**Table 2:** UEFMA reliability and responsiveness test results.

Intraclass Correlation Coefficient					Cronbach Alpha		Concurrent Correlation		Standardized Response Mean	
	ICC ^b^	95% CI		F Test	0.981		FIM	MRS	1.1171	
		Lower Bound	Upper Bound	Sig	Mean	SD	Pearson/ Sig	Pearson/ Sig	95% CI	
									Upper Bound	Lower Bound
Single Measures	0.984 ^a^	0.974	0.990	<0.001	32.750	17.9718	0.789/ <0.001	−0.787/ <0.001	0.9394	1.2695
Average Measures	0.992 ^c^	0.987	0.995	<0.001						

ICC: Intraclass Correlation Coefficient, CI: Confidence Interval, FIM: Functional Independence Measure, MRS: Modified Rankin Scale, Sig.: *p,* ^a^ the estimator is the same, whether the interaction effect is present or not, ^b^ type C intraclass correlation coefficients using a consistency definition-the between- measure variance is excluded from the denominator variance, ^c^ the estimate is computed assuming the interaction effect is absent because it is not estimable otherwise

**Table 3:** CFA (confirmatory factor analysis) model fit indices.

Root Mean Square Residual	Godness of Fit	Baseline Comparisons		Parsimony–Adjusted Measures
RMR	GFI	NFI	RFI	PNFI
0.051	0.980	0.978	0.977	0.911

RMR: Root Mean Square Residual, GFI: Goodness-of-fit Index, NFI: Normed-Fit Index, RFI: Relative Fit Index and PNFI: Parsimonious Normed Fit Index.

Note: Every item corresponds to the numbering on the initial UEFMA scale in English.

**Table 4:** Bayesian modelling of UEFMA regression weights values.

	Mean	S.E.	S.D.	C.S.	Median	95% Lower Bound	95% Upper Bound	SkewNess	Kurtosis	Min	Max
Regression Weights											
AIV. 2 ← UE	0.906	0.005	0.122	1.001	0.904	0.678	1.154	0.201	0.100	0.514	1.420
D 3 ← UE	0.747	0.005	0.105	1.001	0.742	0.552	0.961	0.218	0.118	0.407	1.175
AII.8 ← UE	1.067	0.005	0.119	1.001	1.061	0.847	1.324	0.288	0.199	0.604	1.617
AII.4 ← UE	0.601	0.004	0.104	1.001	0.597	0.404	0.814	0.150	0.159	0.165	1.104
C3d ← UE	0.960	0.006	0.134	1.001	0.955	0.711	1.242	0.239	0.308	0.474	1.552
B2 ← UE	1.185	0.007	0.136	1.001	1.179	0.924	1.474	0.217	0.250	0.720	1.728
AIII.2 ← UE	0.972	0.006	0.124	1.001	0.969	0.736	1.230	0.281	0.619	0.552	1.595
C3.e ← UE	0.989	0.006	0.138	1.001	0.985	0.730	1.271	0.124	0.376	0.412	1.578
D2 ← UE	0.923	0.007	0.144	1.001	0.919	0.650	1.222	0.110	0.233	0.308	1.447
C3c← UE	0.739	0.004	0.119	1.001	0.735	0.516	0.981	0.175	0.107	0.323	1.258
C3a← UE	0.954	0.008	0.142	1.002	0.948	0.692	1.248	0.223	0.047	0.430	1.512
AII.2 ← UE	0.671	0.004	0.114	1.001	0.669	0.458	0.905	0.114	-0.070	0.292	1.084
AII.3← UE	0.594	0.005	0.102	1.001	0.591	0.400	0.801	0.179	0.353	0.188	1.027
AII.6 ← UE	1.088	0.005	0.129	1.001	1.081	0.850	1.355	0.233	0.202	0.626	1.629
AII.5 ← UE	1.088	0.006	0.128	1.001	1.083	0.850	1.353	0.198	0.033	0.633	1.571
AII.7 ← UE	1.063	0.004	0.123	1.001	1.061	0.826	1.316	0.176	0.372	0.607	1.586
AII.9 ← UE	1.145	0.006	0.125	1.001	1.138	0.921	1.406	0.359	0.321	0.737	1.691
AIII.1 ← UE	0.787	0.007	0.130	1.002	0.780	0.553	1.071	0.319	0.299	0.300	1.330
AIII.3 ← UE	1.006	0.005	0.121	1.001	1.000	0.788	1.259	0.339	0.298	0.631	1.571
AIV.1 ← UE	0.746	0.004	0.101	1.001	0.741	0.565	0.964	0.472	0.816	0.408	1.244
AIV.3 ← UE	1.014	0.005	0.129	1.001	1.013	0.766	1.277	0.118	0.107	0.543	1.474
B1 ← UE	1.198	0.007	0.134	1.001	1.193	0.946	1.484	0.238	0.313	0.731	1.788
B3 ← UE	1.036	0.006	0.125	1.001	1.032	0.804	1.299	0.218	-0.013	0.628	1.498
B4 ← UE	1.040	0.005	0.120	1.001	1.035	0.817	1.293	0.273	0.219	0.619	1.598
B5 ← UE	0.747	0.007	0.118	1.002	0.741	0.538	0.998	0.354	0.259	0.359	1.225
C1 ← UE	1.164	0.005	0.123	1.001	1.159	0.942	1.421	0.274	0.132	0.752	1.655
C2 ← UE	1,178	0.005	0.121	1.001	1.172	0.957	1.439	0.401	0.617	0.729	1.749
D1 ← UE	0,858	0.005	0.142	1.001	0.851	0.595	1.148	0.220	0.044	0.351	1.492
C3B ← UE	0,878	0.006	0.126	1.001	0.877	0.644	1.130	0.174	-0.009	0.484	1.401

S.E.: Standard Error, S.D.: Standard Deviation, C.S.: Convergence Statistics, UE: Upper Extremity.

Note: Each item corresponds to the numbering on the initial UEFMA scale in English.

## Supplementary Materials

The following are available online at https://www.mdpi.com/1010-660X/56/8/409/s1, The Romanian version of UEFMA.
