# Supplementary material for: Equal Opportunities for Stroke Survivors’ Rehabilitation: A Study on the Validity of the Upper Extremity Fugl-Meyer Assessment Scale Translated and Adapted into Romanian
Source: Medicina (Kaunas). 2020 Aug 13;56(8):409. doi: 10.3390/medicina56080409 (PMC7466310; doi:10.3390/medicina56080409)
Supplement: Supplementary file 1 [file medicina-56-00409-s001.pdf]

PROTOCOL EFM-MS-RO
Universitatea Transilvania din
Brașov-Facultatea de Medicină

Traducerea versiunii originale FMA, Universitatea Gotemburg, Suedia
www.neurophys.gu.se/sektioner/klinisk-neurovetenskap/forskning/rehab_med/fugl-meyer

EVALUAREA FUGL MEYER MEMBRUL SUPERIOR (FMMS)
(EFM-MS)

Identitate:
Dată:
Examinator:

Fugl-Meyer AR, Jääskö L, Leyman I, Olsson S, Steglind S. The post-stroke hemiplegic patient. A method for evaluation of physical performance. Scandinavian Journal of Rehabilitation Medicine 1975, 7:13-31.

**A. EXTREMITATE SUPERIOARĂ,** poziție șezândă		
**I. Activitate reflexă**	deloc	Pot fi mobilizați
**Flexori:** biceps si flexori de degete (cel puțin unul)	0	2
**Extensori:** triceps	0	2
Subtotal I *(max 4)*		

**II. Mișcarea voluntara în cadrul sinergiilor,** fără ajutor gravitațional			Deloc	Parțial	Total
**Sinergia flexiei:** Mâna de la genunchiul contra lateral la urechea ipsilaterală, *cu palma în sus.*<br>De la sinergia extensorilor (adducție umăr/ rotația internă, extensia cotului, pronația antebrațului) la sinergia flexorilor (abducția umerilor / rotația externă, flexia cotului, supinația antebrațului).	Umăr	retracție	0	1	2
		ridicare	0	1	2
		abducție-90º	0	1	2
		rotație externă	0	1	2
	Cot	flexie	0	1	2
	Antebraț	supinație	0	1	2
**Sinergia extensorilor:** Mâna de la urechea ipsilaterală la genunchiul contra lateral.	Umăr	adducție/rotație internă	0	1	2
	Cot	extensie	0	1	2
	Antebraț	pronație	0	1	2
Subtotal II (max 18)					

**III. Mișcarea voluntară care amestecă sinergiile**, fără compensare		Deloc	Parțial	Total
**Mână-coloana lombară**	nu poate fi efectuată, în fața SIAS	0		
	mână în spatele SIAS (fără compensare)		1	
	la nivelul coloanei vertebrale lombare (fără compensare)			2
**Flexie**<br>Umăr 0-90º,<br>cot la 0º, prono-supinație-0º	abducția imediată sau flexia cotului	0		
	abducție sau cot în flexie în timpul mișcării		1	
	flexie completă la 90 °, menține 0 ° în cot			2
**Prono-supinație**<br>Cot la 90º, Umăr 0º	fără pronație/ supinație, poziția de plecare imposibilă	0		
	pronație / supinație limitată, menține poziția		1	
	pronație completă/ supinație, menține poziția			2
Subtotal III (max 6)				

**IV. Mișcări volitive cu sinergie puțină sau deloc**		Deloc	Parțial	Total
**Abducția umărului**-0-90º<br>Cot la 0º<br>Antebraț pronat	supinația imediată sau flexia cotului	0		
	supinație sau cot în flexie în timpul mișcării		1	
	abducție de 90°, menține extensia și pronația			2
**Flexia umărului** la 90-180º<br>Cot la 0º<br>Prono-supinație 0º	abducție imediată sau flexia cotului	0		
	abducție sau flexia cotului în timpul mișcării		1	
	flexie completă, fără abducție de umăr, menține extensie cot			2
**Prono/supinație**<br>Cot la 0º<br>*Umăr în flexie de 30º-40º*	fără pronație/supinație, poziția de plecare imposibilă	0		
	prono/supinație limitată, menține extensia		1	
	pronație/supinație completă, menține extensia cotului			2
Subtotal IV (max 6)				

**V. Activitatea reflexă normală** (este evaluată numai dacă scorul total de 6 puncte obținut pe partea IV)		Deloc	Parțial	Total
biceps, triceps, flexor degete	0 puncte pe partea a IV-a sau 2 din 3 reflexe puternic hiperactive	0		
	1 reflex puternic hiperactiv sau cel puțin 2 reflexe pline de viață		1	
	maxim 1 reflex animat, nici unul hiperactiv			2
Subtotal V (max 2)				
TOTAL A(max 36)				

**B. PUMNUL-** suportul poate fi asigurat la cot pentru a lua sau ține poziția, fără sprijin la încheietura mâinii, verificați intervalul pasiv de mișcare înainte de testare		Deloc	Parțial	Total
**Stabilitatea la 15 °** dorsiflexie<br>cot la 90°, antebraț pronat<br>umăr la 0 °	mai puțin de 15° dorsiflexie activă<br>dorsiflexia de 15°, nu se face nici o rezistență<br>menține poziția împotriva rezistenței	0	1	2
**Dorsiflexie repetată** / flexie volară<br>cot la 90°, antebraț pronat<br>umăr la 0°, flexie ușoară a degetelor	nu se poate realiza în mod voluntar<br>domeniu limitat de mișcare activ<br>întreaga gamă activă de mișcare, fără probleme	0	1	2
**Stabilitatea la 15 °** dorsiflexie<br>cot la 0°, antebraț pronat<br>*Flexie si abducție de umăr de 20°-30*	mai puțin de 15° dorsiflexie activă<br>dorsiflexia de 15°, fără rezistență<br>menține poziția împotriva rezistenței	0	1	2
**Dorsiflexie repetată** / flexie volară<br>cot la 0°, antebraț pronat<br>*Flexie si abducție de umăr de 20°-30°*	nu se poate realiza în mod voluntar<br>domeniu limitat de mișcare activă<br>întreaga gamă activă de mișcare, fără probleme	0	1	2
**Circumducție**	nu se poate realiza în mod voluntar<br>mișcare jignitoare sau incompletă<br>completă și ușoară	0	1	2
	TOTAL B (max 10)			
**C. MÎINI-**suportul poate fi prevăzut la cot pentru a păstra flexia de 90 °, fără suport la încheietura mâinii, comparați cu mâna neafectată, obiectele sunt interpuse, înțelese activ		Deloc	Parțial	Total
**Flexia degetelor simultan**<br>De la extensie maximă activă/pasivă		0	1	2
**Extensia degetelor simultan**<br>De la flexie maxima activă/pasivă		0	1	2
**PREHENSIUNEA** (prinderea), punctele b,c,d,e se realizează cu antebrațul în supinație si cu comanda :*Ține și nu mă lăsa să iau!*				
**a. Flexia in IDP și IFD** (degetele II-V)<br>Extensia de MCF (degetele II-V)<br>*(rezistență pe extensia MCF)*	nu poate fi efectuată<br>poate deține poziția dar este slab<br>menține poziția împotriva rezistenței	0	1	2
**b. Adducția policelui-**Primul CMC, MCF, IF-0º, tinerea unei foi între police și a 2-a MTF *index*	nu poate fi efectuată<br>poate ține hârtia, dar nu împotriva sustragerii<br>poate ține hârtie împotriva sustragerii	0	1	2
**c. Opozabilitatea**- pulpa policelui- si pulpa degetului II-menținere pix, *în supinație*	nu poate fi efectuată<br>poate ține pixul, dar nu împotriva sustragerii<br>poate ține pixul împotriva sustragerii	0	1	2
**d. Priza cilindrică**<br>Prinderea unui obiect cilindric , opozabilitate cu degetele 2 și 3	nu poate fi efectuată<br>poate ține cilindrul, dar nu împotriva sustragerii<br>poate ține cilindrul împotriva sustragerii	0	1	2
**e. Priza sferică**<br>Degetele în abducție, flexie, police în opoziție-minge de tenis	nu poate fi efectuată<br>poate ține mingea, dar nu împotriva sustragerii<br>poate ține mingea împotriva sustragerii	0	1	2
	TOTAL C (max 14)			
**D. COORDONAREA / VITEZĂ-** șezând, se face cu ambele membre superioare, cu ochi închiși, cu vârful indexului de la genunchi la nas, de 5 ori, cât mai rapid posibil		Marcat	Ușor	Deloc
**Tremor**		0	1	2
**Dismetrie**	pronunțată/nesistematică<br>ușoară și sistematică<br>fără dismetrie	0	1	2
**Timp**		**>5 s**	**2-5 s**	**<1s**
se începe și se termină cu mâna pe genunchi	6 secunde sau mai mult comparativ cu partea sănătoasă 2-5 secunde mai mult față de partea sănătoasă<br>mai puțin de 2 secunde diferență	0	1	2
	**TOTAL D** (max 6)			

**TOTAL A-D** (max 66)	

**H. Sensibilitate-** membrul superior, cu ochii închiși, comparativ cu partea neafectată		**Anestezie**	**Hipoestezie**	**Normal**
**Atingere ușoară**	brațul superior, antebrațul<br>suprafața palmară a mâinii	0<br>0	1<br>1	2<br>2
		absentă<br>mai puțin de 3/4 corect	3/4 corect<br>diferența considerabilă	corect 100%<br>diferență puțină sau deloc
**Poziție**-mici modificări în poziție	umăr<br>cot<br>încheietura<br>degetul mare (articulația IF)	0<br>0<br>0<br>0	1<br>1<br>1<br>1	2<br>2<br>2<br>2
			**TOTAL H** (max 12)	

**I. MOBILITATE PASIVĂ-**membrul superior				**J. DURERE ARTICULARĂ** pe parcursul mobilizării pasive a membrului superior		
Poziția - **șezând,** comparați cu partea neafectată	doar câteva grade (>10 ° în umăr)	Scăzută	Normală	durere constantă pronunțată în timpul sau la sfârșitul mișcării	Durere oarecare	Fără durere
**Umăr**-Flexie(0-180º)<br>Abducție(0-90º)<br>Rotație Externă<br>Rotație Internă	0<br>0<br>0<br>0	1<br>1<br>1<br>1	2<br>2<br>2<br>2	0<br>0<br>0<br>0	1<br>1<br>1<br>1	2<br>2<br>2<br>2
**Cot**-Flexie<br>Extensie	0<br>0	1<br>1	2<br>2	0<br>0	1<br>1	2<br>2
**Antebraț**-Pronație<br>Supinație	0<br>0	1<br>1	2<br>2	0<br>0	1<br>1	2<br>2
**Pumn**-Flexie<br>Extensie	0<br>0	1<br>1	2<br>2	0<br>0	1<br>1	2<br>2
**Degete**-Flexie<br>Extensie	0<br>0	1<br>1	2<br>2	0<br>0	1<br>1	2<br>2
**Total**-max 24				**Total**-max 24		

**A. EXTREMITATE SUPERIOARĂ**	**/36**
**B.PUMN**	**/10**
**C. MÂNĂ**	**/14**
**D. COORDONARE/VITEZĂ**	**/6**
**TOTAL A-D (funcție motorie)**	**/66**

**H.SENSIBILITATE**	**/12**
**J. MOBILITATE PASIVĂ**	**/24**
**J. DURERE ARTICULARĂ**	**/24**
